# Impact of Glycine Supplementation on Growth and Hematological Indices in Florida Pompano (*Trachinotus carolinus*)

**DOI:** 10.1155/anu/8641915

**Published:** 2025-07-24

**Authors:** Trinh H. V. Ngo, Marty Riche, Timothy J. Bruce, Luke A. Roy, D. Allen Davis

**Affiliations:** ^1^School of Fisheries, Aquaculture and Aquatic Sciences, Auburn University, Auburn, Alabama, USA; ^2^Harbor Branch Oceanographic Institute, Florida Atlantic University, Fort Pierce, Florida, USA

**Keywords:** amino acid nutrition, dietary supplementation, Florida pompano, lysozyme activity, serum biochemistry

## Abstract

With the rising incorporation of alternative protein sources in fish diets, understanding amino acid (AA) supplementation strategies, including glycine, optimizes fish growth performance and immune function. A 12-week feeding trial evaluated the effect of dietary glycine (Gly) supplementation on the growth performance and blood biochemistry of juvenile Florida pompano (*Trachinotus carolinus*; 19.55 ± 0.32 g). Experimental diets incorporated soybean meal (SBM), poultry by-product meal (PBM), and corn protein concentrate, and were formulated to contain 40% crude protein and 8% crude lipid. Glycine was supplemented at 0%, 0.25%, 0.5%, and 1%, with alanine (Ala) adjustments to ensure all diets were isonitrogenous. Growth metrics, including final weight (FW) (85.21–90.93 g), weight gain (WG) (336%–366%), and feed conversion ratio (FCR) (1.61–1.69), showed no significant differences among treatments. A significant linear decrease in the hepatosomatic index (*R*^2^ = 0.244, *p* = 0.027), stable liver enzymes, and a significant increase in serum cholesterol at 1% glycine supplementation (*R*^2^ = 0.507; *p* = 0.002) suggest that glycine may influence hepatic metabolism, potentially through enhanced bile acid conjugation. Whole-body proximate composition and AA profiles remained unchanged, and serum lysozyme activity (SLA) showed no significant variation across treatments (*p* = 0.730). These findings suggest that glycine supplementation did not enhance growth but influenced some metabolic parameters. Further research is suggested to clarify the underlying mechanisms under various cultural conditions and stress challenges.

## 1. Introduction

The rising dependence on plant-based ingredients in aquaculture represents a significant strategy to minimize reliance on fishmeal and improve environmental sustainability [1, 2]. This transition presents nutritional challenges for understudied marine species, including Florida pompano (*Trachinotus carolinus*), which are naturally adapted to high-quality animal-based protein sources [3]. Native to the western Atlantic and Gulf coasts, Florida pompano represents a valuable marine finfish with substantial potential for commercial aquaculture development across the United States [4, 5]. Its capacity to perform well in various salinity levels and environmental variables makes it a strong candidate for sustainable aquaculture expansion [6]. Previous studies have shown that when properly supplemented, alternative protein sources, such as soybean meal (SBM) [7, 8] and corn gluten [9], can partially substitute fishmeal in pompano diets. However, plant proteins frequently exhibit imbalanced amino acid (AA) profiles, and may lack essential functional AAs necessary for optimal growth and physiological performance [10]. Insufficient AA nutrition may result in reduced health, decreased growth efficiency, and impaired immune responses [11, 12]. Therefore, developing balanced diet formulations that improve both performance and welfare in fish fed plant-based diets remains a key objective in aquaculture nutrition research, with glycine emerging as a promising candidate due to its unique roles in metabolism and stress response.

Glycine, traditionally classified as a nonessential AA (NEAA) in fish nutrition, is gaining recognition as conditionally essential, especially under certain dietary restrictions or environmental stressors [13]. Collagen comprises approximately one-third of this component, which is necessary for developing and maintaining connective tissues [14]. Glycine also contributes to glutathione synthesis, a critical intracellular antioxidant composed of glycine, cysteine, and glutamate [15], which helps maintain oxidative balance and enhance immune responses in aquatic species [16, 17]. Moreover, glycine contributes to ammonia detoxification pathways by serving as a substrate for glutamate synthesis since glutamate is a precursor in urea cycle activities [18, 19]. Research on various species, such as largemouth bass (*Micropterus salmoides*) [17], Nile tilapia (*Oreochromis niloticus*) [16], and Pacific white shrimp (*Litopenaeus vannamei*) [20], indicates that dietary glycine supplementation improves growth performance, antioxidant capacity, and osmotic stress resistance. However, the physiological response to glycine varies by species, as evidenced by inconsistent results observed in common carp (*Cyprinus carpio*), highlighting differences in glycine metabolism and requirements across diverse taxa [21, 22].

Despite its established roles, the nutritional effects of dietary glycine supplementation have not been evaluated in Florida pompano (*Trachinotus carolinus*), making it an ideal model due to its specific dietary requirements, such as high protein needs and its untapped potential for expanded aquaculture production. This research examined the impact of different levels of dietary glycine supplementation on growth performance and hematological parameters in Florida pompano, aiming to better understand its potential contributions to physiological functions beyond protein synthesis. The hypothesis suggests that moderate dietary glycine supplementation could enhance growth performance by optimizing protein utilization, while also modulating blood biochemical indicators especially increasing immune markers, like lysozyme activity and adjusting hepatic markers, without negatively affecting overall metabolic balance.

## 2. Materials and Methods

### 2.1. Experimental Diets

The basal diet was formulated to be iso-nitrogenous and iso-lipidic (40% protein and 8% lipids), composed of 14% poultry by-product meal (PBM), 48.5% solvent-extracted SBM, and 6.68% corn protein concentrate. Three experimental diets were derived from the basal diet containing 2.21% glycine. Each treatment diet was supplemented with an additional 0.25%, 0.5%, and 1% of glycine (basal, Gly 0.25%, Gly 0.5%, and Gly 1%, respectively), to replace L-alanine based on published evidence from other aquaculture species [16, 17, 22–25]. Diets were prepared using the method described by Corby et al. [26], which involved mixing pre-ground dry ingredients and oil in a food mixer (Hobart, Troy, Ohio, USA) for 15 min. An appropriate amount of boiling water (400 mL kg^−1^) was then blended into the mixture to attain a consistency suitable for pelleting. The prepared moist mash of each diet was extruded through a 3 mm die using a meat grinder. The resulting pellets were then dried in a forced-air oven below 45°C until their moisture content dropped below 10%. Once dried, diets were stored at −20°C in sealed plastic bags until used. Each diet was ground and sieved before feeding to obtain the desired particle size. The University of Missouri Agricultural Experiment Station Chemical Laboratories (Columbia, Missouri, USA) analyzed the proximate and AA composition of the diets (Tables 1 and 2, respectively).

### 2.2. Growth Trial

Florida pompano juveniles (~1 g) were obtained from Trout Lodge Marine Farms LLC (Proaquatix) in Vero Beach, Florida, USA, and reared in an indoor recirculating aquaculture system at the School of Fisheries, Aquaculture, and Aquatic Sciences at Auburn University (Auburn, Alabama, USA). Throughout the nursery phase, fishes were fed to apparent satiation with a 1.5 mm pelleted commercial diet (FF Starter, Zeigler Bros. Inc., Gardner, Pennsylvania, USA), formulated to contain 55% crude protein and 15% crude lipid, until they reached an appropriate size around 19 g. The growth trial was conducted in a recirculating system with 20 culture tanks, water pumps, supplemental aeration (using a central line, regenerative blower, and air diffusers), and mechanical and biological filtration. In addition, a big blue bag filter (Pentair Inc., Minneapolis, Minnesota, USA) was installed to help maintain water quality by removing suspended solids and reducing organic load. The experiment followed a completely randomized design, comprising four dietary treatments and five replicate groups of size sorted fishes (mean initial weight 19.55 ± 0.32 g) assigned to each treatment, with 12 fishes stocked in each aquarium. Each diet was then randomly assigned to five replicate aquaria per treatment. Diets were offered to fishes at 3%–6% body weight daily over six times for hand-feedings. The feed ratio was adjusted each week based on growth, observation of the feeding response, water quality, and mortality. Routine system maintenance included partial water exchanges, backwashing, adding sodium bicarbonate to maintain alkalinity and pH, and siphoning solid waste at the central reservoir as needed. To minimize the risk of parasite infection, fishes were treated with chloroquine phosphate (MP Biomedicals, Solon, Ohio, USA) at a concentration of 60 mg L^−1^ as an antimicrobial agent, and then followed by a freshwater dip lasting 1–2 min during sampling [26]. The prophylactic procedure was repeated each time the fishes were handled.

### 2.3. Sample Collection

At the end of the growth trial, fishes from each aquarium were group-weighed, and individuals were counted to calculate final weight (FW), percentage weight gain (WG), feed conversion ratio (FCR), survival (S), thermal-unit growth coefficient (TGC), and apparent net protein retention (ANPR). Three fishes from each tank were randomly selected, individually weighed, and anesthetized using neutral-buffered tricaine methanesulfonate (MS-222) at a concentration of 100 mg L^−1^ (Western Chemical, Inc., Ferndale, Washington, USA). Blood samples were drawn from the caudal vein using a 1.1 mL S-Monovette sterile syringe with a 22-gauge needle and serum clotting activating gel (Sarstedt, Nümbrecht, Germany) and saved for biochemical analysis. In addition, blood sera were placed into soda-lime glass micro-hematocrit capillary tubes (DWK Life Sciences LLC, Millville, New Jersey, USA), which were wax-sealed (Paul Marienfeld GmbH and Co. KG, Lauda-Königshofen, Germany) for hematocrit analysis. Furthermore, these same fishes were then euthanized with buffered MS-222 for liver collection and weighed to calculate the hepatosomatic index.

The following equations were used to calculate each growth parameter:

  $$\begin{array}{c} \text{Final weight }\left(\text{FW};g\right)=\frac{\text{Total biomass }\left(g\right)}{\text{Final fish number}}, \end{array}$$

  $$\begin{array}{c} \text{Percentage weight gain }\left(\text{WG};\%\right)=\;\frac{\text{Final weight }\left(g\right)-\text{Initial weight }\left(g\right)}{\text{Initial weight }\left(g\right)}\times 100, \end{array}$$

  $$\begin{array}{c} \text{Survival }\left(S;\%\right)=\;\frac{\text{Final fish number at the end of trial}}{\text{Initial fish number at the start of study}}\times 100, \end{array}$$

  $$\begin{array}{c} \text{Thermal-unit growth coefficient }\left(\text{TGC}\right)=\;\frac{{\text{Final weight}}^{\frac{1}{3}}-{\text{Initial weight}}^{\frac{1}{3}}}{\text{Temperature }\left(\;{}^{\circ}C\right)\times \text{Days}}\;\times 100, \end{array}$$

  $$\begin{array}{c} \text{Feed conversion ratio }\left(\text{FCR}\right)=\;\frac{\text{Total feed consumed }\left(g\right)}{\text{Weight gain }\left(g\right)}\times 100, \end{array}$$

  $$\begin{array}{c} \text{ANPR }\left(\%\right)=\;\frac{\text{FW }\left(g\right)\times \text{Final protein content }\left(\%\right)-\text{IW }\left(g\right)\times \text{Initial protein content }\left(\%\right)\;}{\text{Protein intake }\left(g\right)}\times 100, \end{array}$$

  $$\begin{array}{c} \text{Hepatosomatic index }\left(\text{HSI},\%\right)=\;\frac{\text{Weight of liver }\left(g\right)\;}{\text{Body weight }\left(g\right)}\times 100. \end{array}$$

### 2.4. Body Composition Analysis

At the beginning of the trial, 10 fishes were randomly selected from the initial stock population for the initial whole-body composition. After 12 weeks of feeding, three fishes from each tank were randomly collected and stored at −20°C for final biochemical composition analysis. Before conducting proximate analysis, whole fishes were thoroughly homogenized using a meat grinder (Good Food Equipment Co., Dayton, Ohio, USA) following the procedures outlined by the Association of Official Analytical Chemists [27]. The whole-body pompano's proximate composition and AA profile were analyzed at the University of Missouri Agricultural Experiment Station Chemical Laboratories (Columbia, Missouri, USA).

### 2.5. Hematocrit Analysis

Hematocrit values were measured by sealing blood samples in wax-sealed capillary tubes and centrifuged them for 5 min using an IEC Clinical Centrifuge (International Equipment Co., Needham Heights, Massachusetts, USA) at the specified instrument setting. After centrifugation, hematocrit testing was then performed with a micro-capillary reader (International Equipment Co., Needham Heights, Massachusetts, USA).

### 2.6. Serum Biochemistry and Lysozyme Analysis

After bleeding, samples were centrifuged at 4000 × *g* for 10 min to collect serum. Three serum samples from each tank were then pooled into one 450 μL composite sample in sterile 1.5 mL microcentrifuge tubes and stored at −80°C until analysis. The serum biochemical parameters were analyzed by WellFish Tech (Charlottetown, Prince Edward Island, Canada).

The serum lysozyme activity (SLA) was assayed based on a turbidimetric method [28]. Briefly, a standard curve was prepared by diluting a 480 µg mL^−1^ stock solution of chicken egg white lysozyme (Rockland Immunochemicals, Pottstown, Pennsylvania, USA) in sodium phosphate buffer (SPB; 0.04 M Na_2_HPO_4_, pH 6.0) to achieve final concentrations ranging from 0 to 16 µg mL^−1^. Freeze-dried *Micrococcus lysodeikticus* (Worthington Biochemical, Lakewood, New Jersey, USA) was resuspended in SPB at a concentration of 0.25 mg mL^−1^. For each reaction, 250 µL of the bacterial suspension was added to a well, followed by 10 µL of the serum sample. All samples were analyzed in duplicate. After incubation at 37°C for 20 min, absorbance was measured at 450 nm using a Synergy HTX Multimode Reader (BioTek, Winooski, Vermont, USA). SLA was determined by comparing the absorbance values of samples to those of the standard curve.

### 2.7. Water Quality

Water quality parameters, including temperature, dissolved oxygen, and salinity, were monitored twice daily using a YSI Pro 2030 Meter (Yellow Springs Instrument Co., Yellow Springs, OH, USA). Total ammonia nitrogen (TAN) and nitrite were measured biweekly using a YSI EcoSense 9500 Photometer (YSI, Yellow Springs, OH, USA), while pH was measured using a Hanna HI-98107 pHep pH meter (Hanna Instruments, Smithfield, RI, USA).

### 2.8. Statistical Analysis

Prior to conducting statistical analyses, the assumptions of normality and homogeneity of variance were evaluated using the Shapiro–Wilk and Bartlett's tests, respectively [29, 30]. A one-way analysis of variance (ANOVA) was employed to determine significant differences among dietary treatments, and Tukey's Honest Significant Difference (HSD) test was used for pairwise comparisons of treatment means. All statistical analyses were conducted using the SAS system for Windows (V9.4, SAS Institute, Cary, NC). The relationships between the inclusion levels of glycine and FW, WG, ANPR, and glycine retention were plotted and visualized using R 4.3.3 [31] and RStudio 2023.12.1.402 [32] with 95% confidence intervals. A significance level of *α* = 0.05 was used for all statistical analyses.

## 3. Results

### 3.1. Water Quality

Dissolved oxygen concentration averaged 6.75 ± 0.38 mg L^−1^, while the water temperature was maintained at 28.53 ± 0.72°C. Salinity was 13.72 ± 0.58 g L^−1^, and pH remained stable at 8.16 ± 0.11. TAN and nitrite nitrogen levels were relatively low, with values of 0.28 ± 0.17 and 0.07 ± 0.04 mg L^−1^, respectively.

### 3.2. Growth Performance

Dietary glycine supplementation did not significantly affect Florida pompano's growth performance, feed efficiency, or protein retention over the 12-week trial (Table 3). The FW ranged from 85.21 g in the basal diet treatment to 90.93 g in the Gly 0.5% treatment, while WG varied from 336% to 366%, with TGC values between 0.91 and 0.99. FCR ranged from 1.61 to 1.69, and survival was above 90%. The HSI values were between 0.64% and 0.74%, and ANPR ranged from 26.18% to 28.79%.

**Figure 1:**
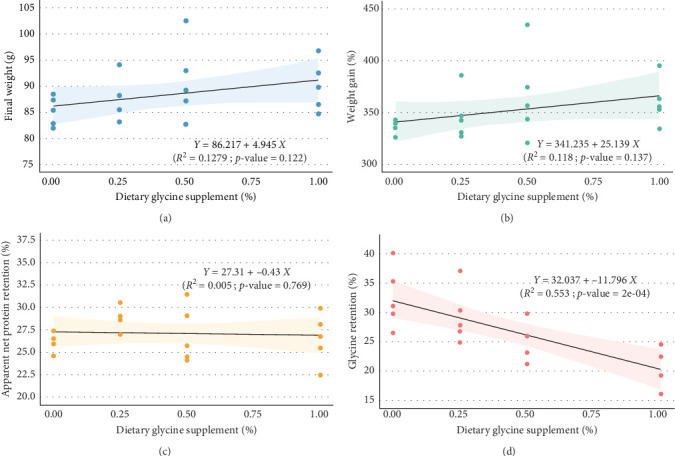
Relationship between various levels of glycine supplementation and growth performance (based on linear regression with 95% confidence interval), including final weight (A), percentage weight gain (B), apparent net protein retention (ANPR) (C), and glycine retention (D) of Florida pompano fed experimental diets with varying levels of glycine for 12 weeks (*n* = 5) in a clear water recirculating system.

Regression analysis (Table 3 and Figure 1) indicated that dietary glycine supplementation had no significant effect (*p*  > 0.05) on most measured growth and utilization parameters in Florida pompano, except for HSI, which showed a significant response to dietary glycine levels (*p* = 0.027, *R*^2^ = 0.244). Linear regression revealed a weak, nonsignificant positive relationship between dietary glycine levels and both FW (*R*^2^ = 0.128, *p* = 0.122) and WG (*R*^2^ = 0.118, *p* = 0.137) over the trial period (Figure 1A,B, respectively). Dietary treatments also showed no significant impact on ANPR (*R*^2^ = 0.005, *p* = 0.769) (Figure 1C). In contrast, a significant linear relationship was observed between glycine supplementation and glycine retention (*R*^2^ = 0.553, *p*  < 0.001) (Figure 1D).

### 3.3. Whole-Body Proximate Composition

Whole-body proximate composition and AA profiles of Florida pompano fed diets with varying levels of glycine supplementation are presented in Table 4. No significant differences (*p*  > 0.05) were observed among dietary treatments for crude protein (17.48%–18.16%), crude fat (7.13%–7.64%), moisture, crude fiber, and ash content. Similarly, whole-body AA composition did not differ significantly among dietary groups (*p*  > 0.05). EAAs, including lysine (1.38%–1.43%), methionine (0.49%–0.51%), threonine (0.73%–0.76%), and arginine (1.15%–1.23%), remained consistent across treatments. NEAAs followed a similar pattern, with glycine ranging from 1.44% to 1.58% and alanine from 1.14% to 1.22%. Total AA content exhibited a slight numerical decrease from 18.21% in the basal group to 17.22% in the Gly 0.5% group, though no significant trends in overall AA composition were observed as a result of glycine supplementation.

### 3.4. Serum Biochemical Indicators and Lysozyme Activity

Glycine supplementation significantly influenced serum cholesterol levels (*p* = 0.005), with fish fed the Gly 1% diet exhibiting the highest cholesterol concentration of 5.04 mmol L^−1^ (Table 5). Regression analysis showed a strong, significant relationship for cholesterol (*R*^2^ = 0.507, *p* = 0.002). In contrast, glycine supplementation did not significantly affect SLA or other serum metabolic parameters, including albumin, alanine aminotransferase (ALT), aspartate aminotransferase (AST), glucose, lactate, creatine kinase, and lactate dehydrogenase (*p*  > 0.05). Likewise, no significant variations were observed in serum electrolytes (sodium, potassium, chloride, calcium, magnesium, phosphate, zinc, and iron) or hematocrit levels across dietary treatments (*p*  > 0.05).

## 4. Discussion

This study evaluated how dietary glycine supplementation affected Florida pompano's growth performance and hematological indices. As a NEAA, glycine is quite important functionally in aquatic species since it helps with osmoregulation, immune modulation, and protein synthesis [33]. Previous studies showed a beneficial effects of glycine supplementation on growth performance, nutrient utilization, and stress resistance in several species, such as hybrid striped bass (*Morone saxatilis* ♀ × *Morone chrysops* ♂), largemouth bass (*Micropterus salmoides*), and red drum (*Sciaenops ocellatus*) at inclusion levels of 2% [17, 34, 35]. Similar advantages have been noted in Nile tilapia (*Oreochromis niloticus*) and common carp (*Cyprinus carpio*) with a reduced inclusion level of 0.5% [16, 22].

To accurately isolate the effects of glycine in this study, L-alanine was used to maintain isonitrogenous conditions across experimental diets. This methodology is a common practice in AA nutrition studies for isolating the effects of a target AA without confounding influences from variations in total dietary nitrogen or overall AA balance [23, 25, 36]. Alanine is a NEAA primarily involved in energy metabolism and gluconeogenesis [13]. Its metabolic pathways do not significantly interfere with glycine-specific metabolic processes or physiological functions [36]. Previous studies support alanine as a neutral balancing AA, unlikely to compete or interact significantly with glycine in terms of metabolism or physiological roles [37]. Therefore, even though the glycine:alanine ratio was altered, it is reasonable to attribute the observed physiological and biochemical outcomes primarily to glycine supplementation.

This study showed that dietary glycine supplementation up to 1% inclusion level (8.18% of dietary protein) throughout a 12-week culture period did not significantly enhance growth performance, FCR, or ANPR in Florida pompano. Survival was more than 90%, with no treatment-related differences. The findings are consistent with previous reports in grass carp (*Ctenopharyngodon idella*) [38] and rainbow trout (*Oncorhynchus mykiss*) [24], which showed that dietary glycine supplementation at 0.5% and 1% (5.70% and 6.75% dietary protein, respectively) did not improve growth metrics. The species-specific character of AA requirements, adequate endogenous glycine synthesis, or environmental conditions linked to the culture system could help explain the lack of notable growth performance improvement in Florida pompano.

Regression analysis revealed no significant correlation between FW or WG and dietary glycine levels, suggesting that the basal diet may already provide enough glycine to reach optimal growth in Florida pompano. The HSI was significantly influenced by dietary glycine levels, with lower HSI values noted in fish that received higher glycine supplementation. This reduction may suggest alterations in hepatic energy storage or lipid metabolism, given that glycine has been demonstrated to influence liver function and improve antioxidant capacity in various species [26]. However, the observed linear decrease in HSI lacks clear biological significance and needs deeper examination, especially regarding its long-term effects on liver function and metabolic regulation in Florida pompano. ANPR showed no response to varying glycine levels, indicating supplemental glycine did not enhance protein utilization efficiency. Higher dietary levels of glycine retention drop to show that excess glycine may either be excreted or redirected to energy pathways, therefore maintaining metabolic balance in line with results from other studies [33, 39, 40].

Previous research has shown that various factors, such as nutritional intake and environmental conditions, can affect the whole-body composition of fish [41, 42]. Glycine supplementation up to 1% did not affect whole-body proximate (moisture, crude protein, crude lipid, crude fiber, and ash) and AA profiles in this study, implying stability in nutrient deposition or AA content [6]. Similar findings were reported by He et al. [25] in hybrid striped bass and by Belghit et al. [24] in rainbow trout, where dietary glycine supplementation did not influence tissue composition or AA profiles. More studies are required to clarify the mechanisms of glycine use and its possible influence on body composition in marine species.

Blood biochemical parameters, which serve as crucial indicators of fish health and physiological responses to dietary modifications [43]. A recent study showed that serum metabolic enzymes (ALT, AST, lactate dehydrogenase, and lipase), glucose concentration or serum electrolyte concentrations (sodium, potassium, and chloride) remained stable with no significant effects by dietary glycine supplementation, suggesting that osmoregulatory function was not disrupted [44]. Consistent hematocrit values across treatments helped to confirm the preservation of erythrocyte volume and circulatory function. While these changes were not statistically significant and might reflect normal biological variability, serum creatine kinase and creatine kinase-myocardial band levels rose numerically [6]. Normal physiological ranges were also preserved in serum levels of necessary minerals, including iron, phosphate, and zinc, which are essential for oxygen transport, enzyme activity, and cellular metabolism [43–45]. These results suggest dietary glycine supplementation did not disrupt mineral absorption and homeostatic regulation.

Notably, glycine supplementation significantly impacted serum cholesterol levels (*p* = 0.005), with fish fed a 1% Gly diet showing the highest levels. The significant strong correlation (*R*^2^ = 0.507, *p* = 0.002) supports the idea that increasing glycine intake may alter lipid metabolism. In teleosts, such as Florida pompano, glycine participates in bile acid conjugation, possibly promoting the production and release of glycocholic acid and related bile salts [45]. Increased bile acid production could help free cholesterol from liver stores, lowering liver lipid content (as shown by a lower HSI) and raise blood cholesterol levels. Song et al. [46] demonstrated that dietary supplementation with cholesterol, taurine, and glycine in spotted seabass (*Lateolabrax maculatus*) influences bile acid metabolism and liver health. The results suggest that even though the basic processes might differ, glycine's role in bile acid conjugation could similarly influence cholesterol levels in different vertebrate species; however, more detailed studies are needed to confirm this.

This study examined the impact of glycine on immune function, specifically through SLA. No significant differences were detected across treatments (*p* = 0.730, ranging from 10.17 to 14.04 μg mL^−1^) under the controlled clear water recirculating system in this study. The observed stability in SLA supports the hypothesis that glycine supplementation does not disrupt metabolic balance. It might also indicate a low immunological challenge within the optimized experimental system, which could hide glycine's immunomodulatory potential [47–49]. Immune function in fish is influenced by the availability and metabolism of AAs [50–52], with emerging evidence indicating a specific role for glycine [13, 53]. However, little is known about how glycine functions in aquatic species, so its precise immunological function remains unclear. The current result agrees with research in Nile tilapia [16], which reported no significant effect of dietary glycine supplementation on SLA, but contrasts with findings by Hoseini et al. [54], who observed increased SLA in beluga and common carp [19], suggesting potential variability due to species or experimental conditions. Still under investigation are the mechanisms of glycine's possible immunomodulatory effects; theories say it boosts antioxidant defenses utilizing glutathione production and regulates inflammation through glycine-gated chloride channels [55]. Glycine's immunological advantages could be context-dependent, depending on environmental conditions, species physiology, baseline immune status, and basal diet composition, according to the results of the current study. Future research should include molecular immune markers and assess glycine supplementation under different stressors or immunological challenges to more fully describe its immunomodulatory effects in Florida pompano and other commercially relevant marine fish species.

## 5. Conclusion

This study's findings suggested that dietary glycine supplementation at levels up to 1% over 12 weeks did not affect growth performance, feed efficiency, overall body proximate composition, or blood biochemistry in Florida pompano. Glycine has been shown to increase serum cholesterol levels, indicating its role in bile acid conjugation within lipid metabolism. SLA exhibited no statistically significant variation among the diets. Given the absence of clear immunological or growth improvements, further research is necessary to establish optimal glycine inclusion rates and to evaluate glycine's metabolic and physiological impacts across various culture environments for Florida pompano.

## Figures and Tables

**Table 1:** Formulation and proximate composition (g 100 g^−1^, as is) of experimental diets used to evaluate the effect of dietary glycine supplementation on the growth of Florida pompano for 12 weeks in a clear water recirculating system.

Composition	Basal	Gly 0.25%	Gly 0.5%	Gly 1%
Poultry by-product meal^a^	14.00	14.00	14.00	14.00
Soybean meal^b^	48.50	48.50	48.50	48.50
Corn protein concentrate^c^	6.68	6.68	6.68	6.68
Fish oil^d^	3.86	3.86	3.86	3.86
Soy oil	1.82	1.82	1.82	1.82
Soy lecithin^e^	0.50	0.50	0.50	0.50
Corn starch^f^	0.24	0.24	0.24	0.24
Whole wheat^g^	20.00	20.00	20.00	20.00
Mineral premix^h^	0.25	0.25	0.25	0.25
Vitamin premix^i^	0.50	0.50	0.50	0.50
Choline chloride^j^	0.20	0.20	0.20	0.20
Stay-C 35%^k^	0.10	0.10	0.10	0.10
CaP-dibasic^l^	1.75	1.75	1.75	1.75
Alanine^m^	1.00	0.75	0.50	0.00
Glycine^m^	0.00	0.25	0.50	1.00
Methionine^m^	0.10	0.10	0.10	0.10
Taurine^m^	0.50	0.50	0.50	0.50
Proximate analysis^n^ (g 100 g^−1^ as is)				
Crude protein	42.70	42.72	43.47	43.56
Moisture	5.22	5.05	4.06	3.70
Crude fat	9.13	9.10	9.12	9.14
Crude fiber	2.60	2.66	2.61	2.71
Ash	7.10	7.09	7.12	7.06

^a^River Valley Ingredients, Hanceville, Alabama, USA.

^b^De-hulled solvent-extracted soybean meal, Bunge Limited, Decatur, Alabama, USA.

^c^Empyreal 75, Cargill Corn Milling, Cargill, Inc, Blair, Nebraska, USA.

^d^Omega Protein Inc., Houston, Texas, USA.

^e^The Solae Company, St. Louis, Missouri, USA.

^f^Ingredi Company, 501 Chesapeake Park Plaza Baltimore, Maryland, USA.

^g^ADM, 4666 E Faries Pkwy, Decatur, Illinois, USA.

^h^Mineral premix (g 100 g^−1^premix): cobalt chloride, 0.008; cupric sulfate pentahydrate, 0.01, ferrous sulfate heptahydrate, 20, manganous sulfate anhydrous, 2.90; potassium iodide, 0.240; sodium selenite, 0.048; zinc sulfate heptahydrate, 17.600, and *α* cellulose 59.194.

^i^Vitamin premix (g kg^−1^premix): thiamin HCL, 8.0; riboflavin, 8.0; pyridoxine HCl, 5.0; Ca-pantothenate, 20.0; niacin, 40.0; biotin, 0.040; folic acid, 1.80; cyanocobalamin, 0.002; vitamin A acetate (500,000 IU g^−1^), 2.40; vitamin D₃ (400,000 IU g^−1^), 0.50; DL-*α*-tocopheryl acetate, 80.0; and *α* cellulose, 834.258.

^j^MP Biomedicals Inc., Solon, Ohio, USA.

^k^Stay C, (L-ascorbyl-2-polyphosphate 35% Active C), Roche Vitamins Inc., Parsippany, New Jersey, USA.

^l^BeanTown Chemical, 9 Sagamore Park Road Hudson, New Hampshire, USA.

^m^Tokyo Chemical Industry, Portland, Oregon, USA.

^n^Analyzed at University of Missouri Agricultural Experiment Station Chemical Laboratories (Columbia, Missouri, USA).

**Table 2:** Proximate and amino acid (AA) analysis^a^ (g 100 g^−1^, as is) used to evaluate the effect of dietary glycine supplementation on the growth of Florida pompano for 12 weeks.

	Basal	Gly 0.25%	Gly 0.5%	Gly 1%
Essential amino acids (EAA)				
Arginine	2.72	2.70	2.71	2.77
Histidine	1.02	1.01	1.01	1.04
Isoleucine	1.92	1.91	1.90	1.95
Leucine	3.69	3.70	3.68	3.80
Lysine	2.40	2.37	2.38	2.43
Methionine	0.83	0.83	0.83	0.85
Phenylalanine	2.15	2.15	2.14	2.21
Threonine	1.58	1.58	1.58	1.62
Tryptophan	0.38	0.41	0.42	0.43
Valine	2.15	2.12	2.12	2.16
Nonessential amino acids (NEAA)				
Alanine	3.19	2.98	2.81	2.33
Aspartic acid	4.01	3.97	3.99	4.06
Cysteine	0.63	0.65	0.63	0.65
Glutamic acid	7.87	7.85	7.88	8.03
Glycine	2.21	2.41	2.72	3.27
Hydroxylysine	0.10	0.09	0.10	0.10
Hydroxyproline	0.28	0.27	0.29	0.28
Lanthionine	0.00	0.00	0.00	0.00
Ornithine	0.04	0.03	0.03	0.04
Proline	2.60	2.58	2.62	2.67
Serine	1.73	1.71	1.75	1.78
Taurine	0.74	0.74	0.73	0.75
Tyrosine	1.58	1.57	1.58	1.61
Total amino acids	43.82	43.63	43.90	44.83

^a^Analyzed at University of Missouri Agricultural Experiment Station Chemical Laboratories (Columbia, Missouri, USA).

**Table 3:** Growth performance of Florida pompano cultured in a clear water recirculating system for 12 weeks when fed dietary glycine levels.

Diets	Final weight (g)	Weight gain (%)	TGC	FCR	Survival (%)	HSI (%)	ANPR (%)
Basal	85.21	336	0.91	1.69	100.00	0.73	26.18
Gly 0.25%	87.30	347	0.94	1.61	95.00	0.74	28.79
Gly 0.5%	90.93	366	0.99	1.62	90.00	0.64	26.96
Gly 1%	90.08	360	0.98	1.63	96.67	0.64	26.55

*p*-Value	0.306	0.316	0.284	0.567	0.131	0.060	0.308
PSE	2.29	13	0.03	0.04	2.83	0.03	1.02

Regression							
*R*^2^	0.128	0.118	0.128	0.034	0.276	0.244	0.005
*p*-Value	0.122	0.137	0.122	0.437	0.065	0.027	0.769

*Note:* Values represent the mean of five replicates of each diet. This means not sharing any letter is significantly different from Tukey's HSD test at the 5% significance level.

Abbreviations: ANPR, apparent net protein retention; FCR, feed conversion ratio; HSI, hepatosomatic index; PSE, pooled standard error; TGC, thermal growth coefficient.

**Table 4:** Proximate and amino analysis^a^ of whole body (g 100 g^−1^, as is) of Florida pompano cultured in a clear water recirculating system for 12 weeks and fed diets supplemented with various levels of glycine.

	Basal	Gly 0.25%	Gly 0.5%	Gly 1%	PSE	*p*-Value
Essential amino acids (EAA)						
Arginine	1.23	1.18	1.15	1.16	0.02	0.065
Histidine	0.43	0.42	0.41	0.42	0.01	0.565
Isoleucine	0.76	0.74	0.73	0.73	0.02	0.774
Leucine	1.25	1.23	1.21	1.21	0.03	0.665
Lysine	1.43	1.40	1.38	1.38	0.03	0.532
Methionine	0.51	0.50	0.49	0.49	0.01	0.250
Phenylalanine	0.72	0.70	0.69	0.69	0.01	0.383
Threonine	0.76	0.75	0.73	0.73	0.01	0.251
Tryptophan	0.15	0.14	0.15	0.15	0.01	0.758
Valine	0.87	0.85	0.83	0.84	0.02	0.539
Nonessential amino acids (NEAA)						
Alanine	1.22	1.19	1.14	1.15	0.02	0.093
Aspartic acid	1.66	1.61	1.58	1.59	0.03	0.320
Cysteine	0.17	0.16	0.16	0.17	0.00	0.612
Glutamic acid	2.43	2.37	2.31	2.33	0.04	0.139
Glycine	1.58	1.52	1.44	1.47	0.07	0.472
Hydroxylysine	0.05	0.05	0.05	0.05	0.00	0.939
Hydroxyproline	0.36	0.35	0.32	0.33	0.03	0.791
Lanthionine	0.03	0.03	0.03	0.03	0.00	0.647
Ornithine	0.03	0.02	0.02	0.02	0.00	0.547
Proline	0.93	0.89	0.85	0.86	0.03	0.368
Serine	0.64	0.63	0.61	0.62	0.01	0.280
Taurine	0.44	0.43	0.42	0.40	0.01	0.065
Tyrosine	0.56	0.54	0.54	0.53	0.01	0.464
Total amino acids	18.21	17.70	17.22	17.36	—	—
Proximate composition (g 100 g^−1^)						
Moisture	71.62	71.53	71.61	71.52	0.43	0.997
Crude protein	17.48	18.16	17.73	17.61	0.31	0.443
Crude fat	7.13	7.40	7.64	7.18	0.42	0.813
Crude fiber	0.10	0.12	0.17	0.06	0.04	0.279
Ash	3.05	3.15	3.14	3.22	0.09	0.652

*Note:* Values represent the mean of five replicates of each diet. This means not sharing any letter is significantly different from Tukey's HSD test (parametric ANOVA) at the 5% level of significance.

^a^Analyzed at University of Missouri Agricultural Experiment Station Chemical Laboratories (Columbia, Missouri, USA).

**Table 5:** Effect of dietary glycine supplementation on the serum metabolic parameters of Florida pompano cultured for 12 weeks in a clear water recirculating system.

Parameters^a^	Basal	Gly 0.25%	Gly 0.5%	Gly 1%	PSE	*p*-Value	Regression	
							*R* ^2^	*p*-Value
Albumin (g L^−1^)	13.94	14.21	13.61	14.76	0.32	0.109	0.122	0.132
Alanine aminotransferase (U L^−1^)	8.00	14.40	5.80	7.40	3.04	0.247	0.027	0.490
Aspartate aminotransferase (U L^−1^)	40.60	61.20	41.20	46.40	11.92	0.596	0.000	0.950
Calcium (mmol L^−1^)	3.89	3.85	3.77	4.03	0.09	0.256	0.201	0.149
Cholesterol (mmol L^−1^)	4.63^ab^	4.53^b^	4.36^b^	5.04^a^	0.12	0.005	0.507	0.002
Creatine kinase (U L^−1^)	2584.20	3828.20	2780.80	3388.80	1085.16	0.842	0.005	0.768
Creatine kinase MB (U L^−1^)	1087.80	1613.00	1134.40	1371.00	406.12	0.787	0.003	0.830
Glucose (mmol L^−1^)	9.36	8.36	7.82	9.54	0.60	0.176	0.257	0.080
Iron (μmol L^−1^)	10.80	12.00	11.00	11.40	1.02	0.848	0.002	0.866
Lactate (mmol L^−1^)	9.32	8.38	7.56	9.50	0.85	0.375	0.166	0.215
Lactate dehydrogenase (U L^−1^)	134.80	200.20	164.40	191.20	53.58	0.823	0.017	0.582
Lipase (U L^−1^)	5.23	5.42	9.58	5.72	2.21	0.474	0.005	0.763
Magnesium (mmol L^−1^)	1.65	1.58	1.51	1.69	0.06	0.255	0.208	0.137
Phosphate (mmol L^−1^)	4.34	4.28	3.95	4.37	0.18	0.356	0.000	0.985
Zinc (μmol L^−1^)	266.48	271.72	265.36	289.92	10.61	0.359	0.123	0.130
Sodium (mmol L^−1^)	191.40	192.40	190.00	192.00	1.60	0.733	0.000	0.958
Potassium (mmol L^−1^)	6.37	6.41	6.14	6.47	0.12	0.297	0.0074	0.717
Chloride (mmol L⁻^1^)	165.60	167.40	165.80	165.20	1.56	0.765	0.013	0.628
Hematocrit (%)	43.07	42.53	45.40	45.13	1.54	0.474	0.082	0.222
Lysozyme activity (μg mL^−1^)	13.24	12.10	14.04	10.17	2.54	0.730	0.035	0.429

*Note:* Values represent the mean of five replicates of each diet. Means not sharing any letter are significantly different by the Tukey's HSD-test at the 5% level of significance.

Abbreviation: PSE, pooled standard error.

^a^Analyzed by WellFish Tech (Charlottetown, Prince Edward Island, Canada). Lysozyme activities were analyzed at Auburn University (Auburn, Alabama, USA).

## Data Availability

The data are available upon request from the authors.
